# The Interruption of Transmission of Human Onchocerciasis by an Annual Mass Drug Administration Program in Plateau and Nasarawa States, Nigeria

**DOI:** 10.4269/ajtmh.19-0577

**Published:** 2020-02-10

**Authors:** Frank O. Richards, Abel Eigege, John Umaru, Barminas Kahansim, Solomon Adelamo, Jonathan Kadimbo, Jacob Danboyi, Hayward Mafuyai, Yisa Saka, Gregory S. Noland, Chukwuma Anyaike, Michael Igbe, Lindsay Rakers, Emily Griswold, Thomas R. Unnasch, B. E. B. Nwoke, Emmanuel Miri

**Affiliations:** 1 The Carter Center, Atlanta, Georgia;; 2 The Carter Center Nigeria, Jos, Nigeria;; 3 Plateau State Ministry of Health, Jos, Nigeria;; 4 Nasarawa State Ministry of Health, Lafia, Nigeria;; 5 University of Jos, Jos, Nigeria;; 6 Department of Public Health, Federal Ministry of Health, Abuja, Nigeria;; 7 University of South Florida, Tampa, Florida;; 8 Imo State University, Owerri, Nigeria

## Abstract

Plateau and Nasarawa states in central Nigeria were endemic for onchocerciasis. The rural populations of these two states received annual ivermectin mass drug administration (MDA) for a period of 8–26 years (1992–2017). Ivermectin combined with albendazole was given for 8–13 of these years for lymphatic filariasis (LF); the LF MDA program successfully concluded in 2012, but ivermectin MDA continued in areas known to have a baseline meso-/hyperendemic onchocerciasis. In 2017, serological and entomological assessments were undertaken to determine if MDA for onchocerciasis could be stopped in accordance with the current WHO guidelines. Surveys were conducted in 39 sites that included testing 5- to < 10-year-old resident children by using ELISA for OV16 IgG4 antibodies, and *Onchocerca volvulus* O150 pooled polymerase chain reaction (PCR) testing of *Simulium damnosum* s.l. vector heads. Only two of 6,262 children were OV16 positive, and none of 19,056 vector heads were positive for parasite DNA. Therefore, both states were able to meet WHO stop-MDA thresholds of an infection rate in children of < 0.1% and a rate of infective blackflies of <1/2,000, with 95% statistical confidence. Transmission of onchocerciasis was declared interrupted in Plateau and Nasarawa states by the Federal Ministry of Health, and 2.2 million ivermectin treatments/year were stopped in 2018. Post-treatment Surveillance was launched focusing on entomological monitoring on borders with neighboring onchocerciasis-endemic states. An apparent positive impact of the LF MDA program on eliminating hypo-endemic onchocerciasis was observed. This is the first stop-MDA decision for onchocerciasis in Nigeria and the largest single stop-MDA decision for onchocerciasis yet reported. This achievement, along with the process used in adapting and implementing the 2016 WHO stop-MDA guidelines, will be important as a potential model for decision makers and national onchocerciasis elimination committees in other African countries that are charged with advancing their programs.

## INTRODUCTION

Human onchocerciasis (“river blindness”) is a parasitic infection caused by the filarial nematode *Onchocerca volvulus*.^1^ In addition to severe eye disease, onchocerciasis causes papular or hypopigmented skin lesions and intense itching. The parasite is transmitted by certain species of *Simulium* blackflies, with the most common vector being *Simulium damnosum* sensu lato (s.l.).^2^ In humans, the adult worms cluster in subcutaneous fibrous onchocercomas (commonly referred to as “nodules”) that are often visible and/or palpable. In these nodules, fertilized females release microfilariae (mf) that migrate in the sub dermis and eye, causing immune reactions that result in the major morbidities associated with the infection. Some mf are picked up when the vector flies take a blood meal. In the flies, the mf eventually develop into the third-stage larvae (L3) that are infectious to humans on subsequent blood meals. In the humans, the larvae then develop into adult worms and so continue the life cycle. There are no known environmental or epidemiologically important animal reservoirs of *O. volvulus*.^3^

Mass drug administration (MDA) with ivermectin (Mectizan^®^, donated by Merck & Co., Inc., Kenilworth, NJ) is the WHO-recommended strategy for the control of onchocerciasis.^4^ Ivermectin is a potent microfilaricide that also has a limited effect on the viability and reproductive capabilities of adult worms, which normally live 8–14 years. Female worms are unable to release their mf for 3–6 months after ivermectin treatment.^5^ This means disease elimination strategies have required repeated once- or twice-per-year MDA cycles for many years until the adult parasite population collapses, thereby permanently interrupting transmission unless the infection is reintroduced. Early models estimated that after about 25 years of annual MDA, the treatment program could be safely stopped.^6^

## HISTORY OF THE IVERMECTIN-BASED MDA PROGRAMS IN PLATEAU AND NASARAWA STATES

**Figure 1.**
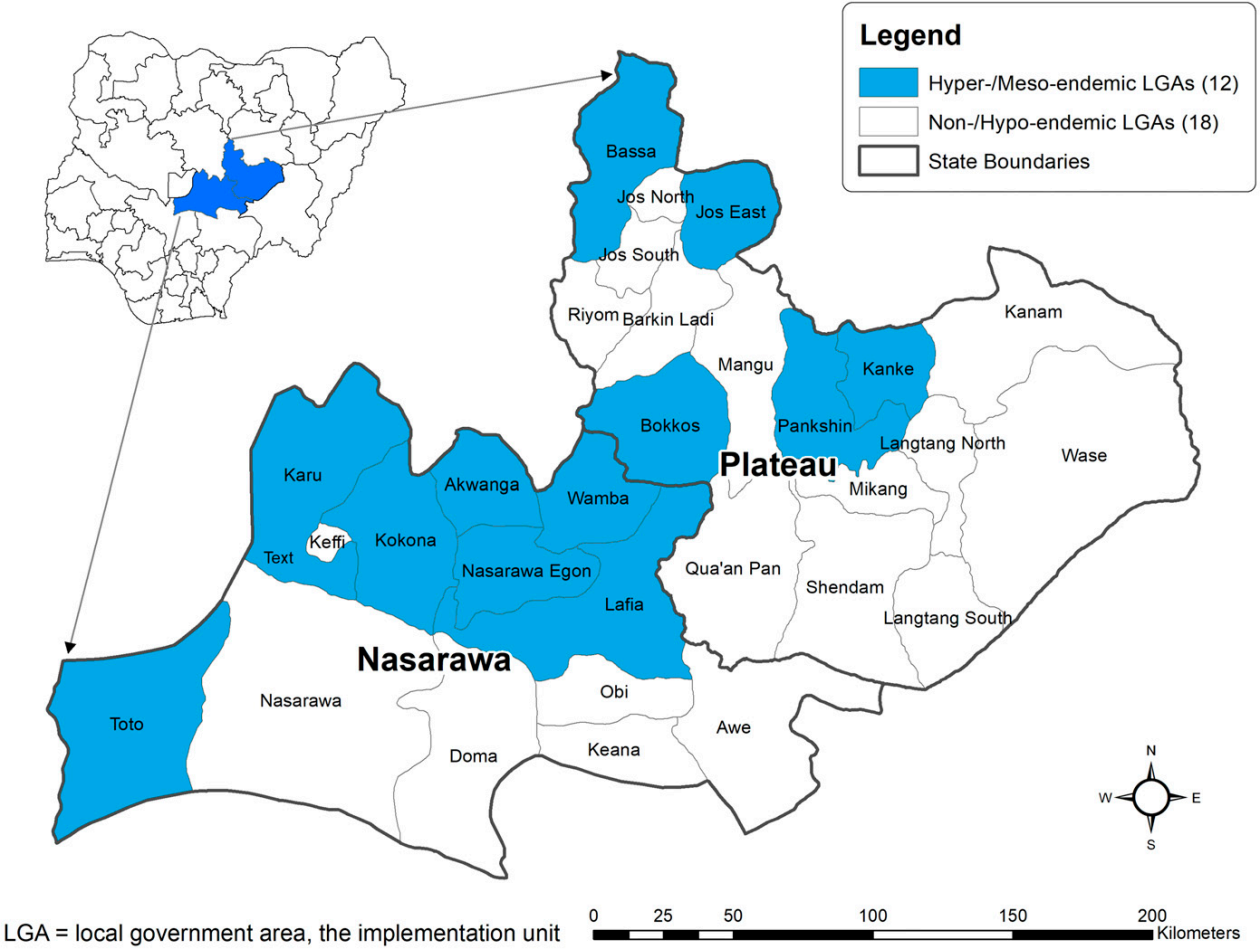
Plateau and Nasarawa states, showing local government areas (LGAs) by baseline endemicity of onchocerciasis and lymphatic filariasis. This figure appears in color at www.ajtmh.org.

Plateau and Nasarawa states are located in central Nigeria and have an estimated 7 million residents, most of whom live in rural agricultural villages. When ivermectin treatments were launched in Plateau state, there were 23 administrative districts (called local government areas [LGAs]). Nasarawa state, originally the eastern part of Plateau state, split off in 1996. There are currently 30 LGAs: 17 in Plateau and 13 in Nasarawa (Figure 1).

### The early onchocerciasis program.

The Onchocerciasis Program began in Plateau state in 1991 with a disease-mapping exercise based on parasitological examinations of convenience samples of 30–50 adult male residents in 108 rural villages scattered throughout the state.^7–9^ Superficial skin biopsies (“skin snips,” weighing 1–3 mg) were obtained from the left and right iliac crest using field-sterilizable corneoscleral punches. The skin snips were incubated in normal saline for 24 hours and then examined by microscopy (×40) for mf. Villages having an mf prevalence of ≥ 5% were considered to be mesoendemic, and any LGA having one such village was considered in need of MDA in its entirety. Hyperendemic LGAs were those having at least one village with mf prevalence of ≥ 80%. When analyzed in 1991, 10 of the original 23 LGAs in Plateau were in need of MDA. After Nasarawa state split from Plateau, seven new LGAs were formed and the final administrative configuration of the two-state area consisted of 12 LGAs classified as mesoendemic or hyperendemic, and thus in need of MDA (Table 1). Eighteen LGAs were either non-endemic or had villages with less than 5% mf prevalence (non- or hypo-endemic, hereafter referred to as “non-/hypo-endemic”) and were left untreated. It should be noted that this skin snip mapping exercise was completed before adoption by Nigeria of the “rapid epidemiological onchocerciasis mapping” (REMO) technique, where onchocerciasis endemicity in the country was classified based on nodule (onchocercoma) rates, as reported by Gemade et al. in 1998.^10^

**Table 1:** Plateau and Nasarawa states: baseline microfilaridermia endemicity, numbers of ivermectin-based treatment rounds, and years of mass drug administration (MDA), by local government area (LGA)

State		LGA	Range of baseline village microfilariae prevalence in skin snips in surveys conducted in 108 villages, 1991–1992 (%)	Onchocerciasis endemicity classification	No. of ivermectin treatment rounds	Years of ivermectin-based MDA
1	Nasarawa	Akwanga	5–100	Hyperendemic	26	1992–2017
2		Awe	0–4	Non-/hypo-endemic	10	2003–2012‡
3		Doma	0–4	Non-/hypo-endemic	11	2002–2012‡
4		Karu	0–79	Mesoendemic	25	1993–2017
5		Keana*	0–4	Non-/hypo-endemic	8	2002–2009‡
6		Keffi†	0–4	Non-hypendemic	8	2002–2009‡
7		Kokona*	30–79	Mesoendemic	25	1993–2017
8		Lafia	0–29	Mesoendemic	25	1993–2017
9		Nasarawa*	0–4	Non-/hypo-endemic	11	2002–2012‡
10		Nasarawa Egon	5–79	Mesoendemic	26	1992–2017
11		Obi	0–4	Non-/hypo-endemic	11	2002–2012‡
12		Toto	5–100	Hyperendemic	26	1992–2017
13		Wamba*	30–79	Mesoendemic	11	2002–2012‡
1	Plateau	Barkin Ladi	0–4	Non-/hypo-endemic	8	2002–2009
2		Bassa	5–79	Mesoendemic	26	1992–2017‡
3		Bokkos	30–79	Mesoendemic	25	1993–2017
4		Jos East*	5–79	Mesoendemic	26	1992–2017
5		Jos North	0–4	Non-/hypo-endemic	8	2002–2009‡
6		Jos South	0–4	Non-/hypo-endemic	11	2002–2012‡
7		Kanam	0–4	Non-/hypo-endemic	11	2002–2012‡
8		Kanke*	5–29	Mesoendemic	25	1993–2017
9		Langtang North	0–4	Non-/hypo-endemic	11	2002–2012‡
10		Langtang South	0–4	Non-/hypo-endemic	8	2003–2009‡
11		Mangu	0–4	Non-/hypo-endemic	11	2002–2012‡
12		Mikang*	0–4	Non-Hypo-endemic	11	2002–2012‡
13		Pankshin	0–29	Mesoendemic	25	1993–2017
14		Qua’an Pan	0–4	Non-/hypo-endemic	11	2002–2012‡
15		Riyom*	0–4	Non-/hypo-endemic	11	2002–2012‡
16		Shendam	0–4	Non-/hypo-endemic	11	2002–2012‡
17		Wase	0–4	Non-/hypo-endemic	11	2002–2012‡

* Indicate LGAs formed after the baseline mapping whose endemicity has been assigned based on values from their parent LGAs’ baseline assessments in 1991. Keana was carved from Obi, Kokona from Keffi,† Wamba from Akwanga, Jos East from Jos South, Kanke and Mikang from Pankshin, and Riyom from Barkin Ladi.

† Keffi (originally mesoendemic) was reduced in size during the partition and made into a purely urban LGA, with Kokona created from the remainder of Keffi. This resulted in major endemicity changes for Keffi going from mesoendemic on original maps to current hypo-endemic classification, on the assumption that urban areas do not ecologically support transmission of onchocerciasis.

‡ MDA provided for lymphatic filariasis with combination therapy with ivermectin–albendazole.

**Figure 2.**
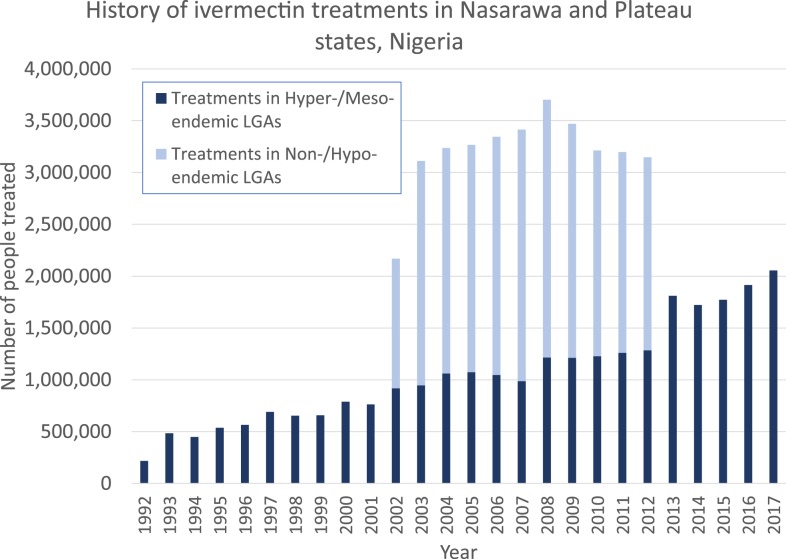
History of ivermectin treatments in Nasarawa and Plateau states, Nigeria. Note: From 2000–2012, the 12 local government areas (LGAs) that were meso-hyperendemic for onchocerciasis (dark bars) were treated with the ivermectin–albendazole combination. After stopping mass drug administration for lymphatic filariasis transmission in 2012, treatments in those 12 LGAs reverted to ivermectin monotherapy. Treatments in the 18 non-endemic or hypo-endemic onchocerciasis LGAs (non-/hypo-endemic) with ivermectin–albendazole occurred only during the 2000–2012 LF campaign (light bars). This figure appears in color at www.ajtmh.org.

Ivermectin-based MDA was launched in the (now) 12 mesoendemic or hyperendemic (“meso-/hyperendemic”) onchocerciasis LGAs in 1992–1993.^7,9^ The program’s treatment coverage goal was to reach at least 80% of the eligible population using community-based distributors selected by the individual communities and trained by Ministry of Health/NGO staff. Community-based distributors were then given 2–4 weeks to complete drug distribution and to report their treatment results back to the Ministry of Health. Treatment coverages were then verified in spot checks by Ministry of Health/NGO staff. By 1994, all communities targeted for treatment were under MDA. In 1995, the program achieved > 80% reported coverage. In 1996, the MDA strategy was successfully reoriented to the “community-directed treatment with ivermectin” strategy of the African Program for Onchocerciasis Control (APOC) and ≥ 80% coverage per year continued to be reported. By the end of 2017, the 12 meso-/hyperendemic LGAs had received between 25 and 26 annual rounds of MDA (Table 1). Overall treatment numbers for the onchocerciasis meso-/hyperendemic LGAs by year are shown in Figure 2 (dark bars).

### Lymphatic filariasis MDA in Plateau and Nasarawa states.

The history of the lymphatic filariasis (LF) program in Plateau and Nasarawa states has been published in detail.^11–15^ Briefly, it began in 1997 with mapping of seroprevalence of circulating LF antigen in adults. All 30 LGAs were found to be greater than the 1% endemicity threshold (Figure 1), with LGA antigen prevalence ranging from 4% to 62%.^12^ Combined ivermectin/albendazole MDA was scaled up over 4 years between 2000 and 2003, beginning in 2000–2001 by “piggy-backing” onto the onchocerciasis MDA programs in the 12 meso-/hyperendemic onchocerciasis LGAs,^11^ and expanding to all 30 LGAs by 2003 (Figure 2, light bars). Overall reported coverage in the two-state area remained > 80% of the treatment-eligible population for all years. In 2003, a coverage survey that included the 12 meso-/hyperendemic onchocerciasis LGAs showed an ivermectin/albendazole treatment coverage (using the total population as the denominator) of 72.2% (95% CI 65.5–79.0).^12^

Various LF evaluations and transmission assessment surveys led to the decision to halt LF MDA in Plateau and Nasarawa states after 2012.^13–15^ As a result, the 18 non-/hypo-endemic onchocerciasis LGAs received a total of 8–11 rounds of ivermectin-based MDA under the LF program (Table 1). Mass drug administration for onchocerciasis ivermectin monotherapy continued in the 12 meso-hyperendemic onchocerciasis LGAs after the LF MDA program came to its successful conclusion.

### Impact assessments for onchocerciasis during the MDA treatment interval.

Assessments in sentinel villages (SVs) were conducted periodically in highly endemic SVs to determine the impact of MDA on onchocerciasis prevalence. Evans et al.^8^ reported a 2009 survey in six sentinel and eight “spot check” villages located in five of the 12 onchocerciasis meso-/hyperendemic LGAs. The results showed the mean skin snip mf prevalence in those villages had dropped by 99% (38% to 0.3%). The survey also tried to meet the two requirements of the WHO for halting MDA: 1) IgG4 antibody to OV16 prevalence among a sample of at least 3,000 children must be < 0.1% (upper 95% confidence limit [UCL]); and 2) rates of infective (L3) larvae in vector heads must be < 1/2,000 (UCL) as determined by O150 PCR, with at least 6,000 *S. damnosum* s.l. having been examined.^16,17^ Unfortunately, the OV16 rates in the Evans study were too high (seven positives among 4,451 children, 0.16% [UCL] 0.32%), and although no PCR positive head pools were found, insufficient (1,568) *S. damnosum* had been captured by human attractant landing captures.

In 2012, an APOC team conducted an independent skin snip assessment in 10 non-SVs in Plateau and Nasarawa states.^18^ Four of these sites were in the Plateau LGAs of Riyom (non-/hypo-endemic), Bokkos (mesoendemic), Mangu (non-/hypo-endemic), and Pankshin (mesoendemic). Six were in Nasarawa: Awe LGA (non-/hypo-endemic), Akwanga (hyperendemic), Nasarawa Egon (mesoendemic), Kokona (mesoendemic), Karu (mesoendemic), and Nasarawa (non-/hypo-endemic). The results showed only 2 mf-positive adults (0.1%) among 1,911 examined (both from the village of Toff in Bokkos LGA in Plateau state). The APOC concluded that the two states passed the preliminary (1a survey) requirement of < 1% skin snip prevalence, but required further skin snips and entomology (PCR in 6,000 flies) be carried out before a “stop-MDA” decision could be made.^19^ In consideration of these 2009 and 2012 surveys, the Federal Ministry of Health (FMOH) determined that despite halting LF MDA in Plateau and Nasarawa states in 2012, MDA with ivermectin for onchocerciasis had to continue in the 12 meso-/hyperendemic LGAs.

### The Nigeria Onchocerciasis Elimination Committee (NOEC).

**Figure 3.**
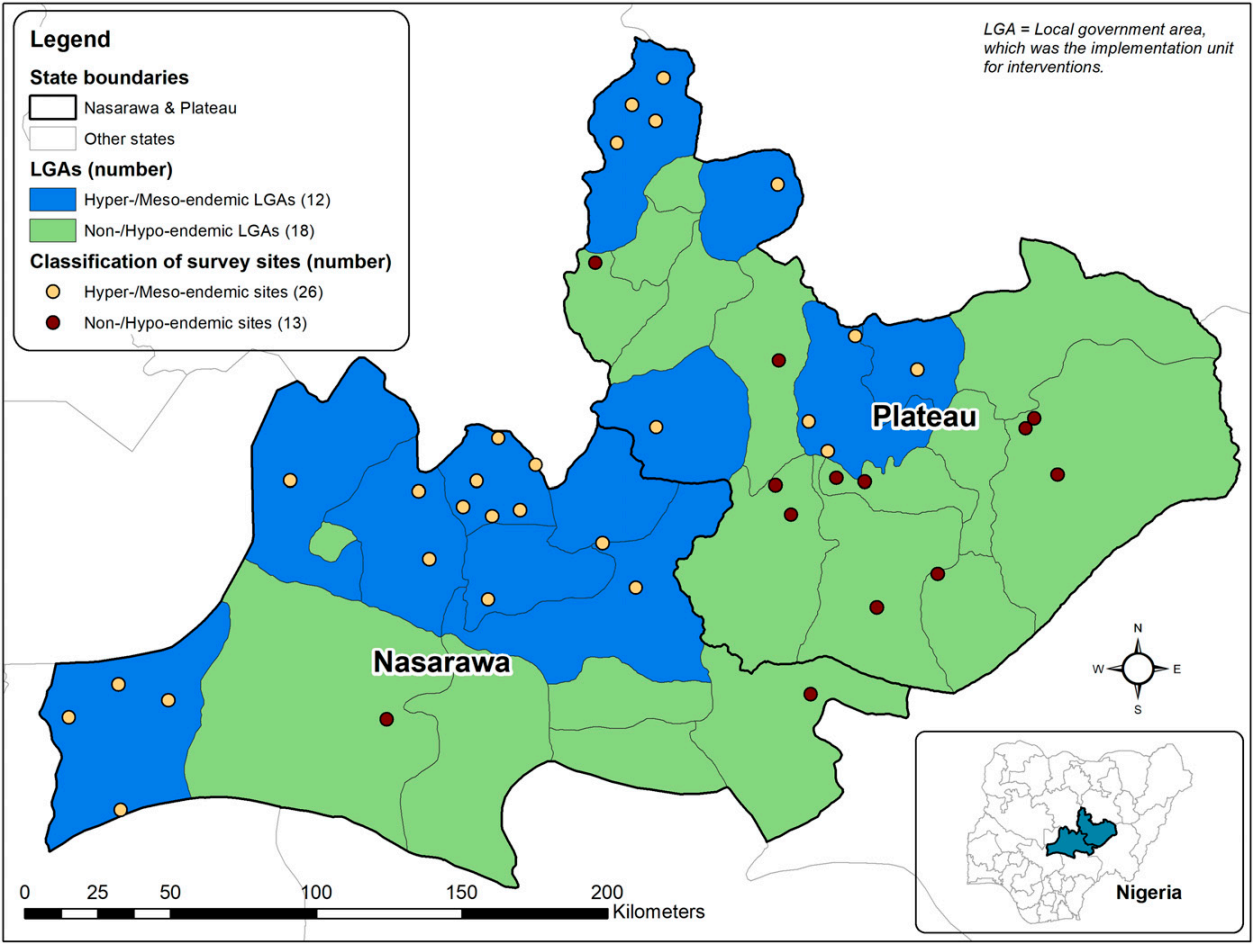
Map of the two-state area showing locations of 2017 survey sites, by baseline LGA endemicity. This figure appears in color at www.ajtmh.org.

In 2015, the NOEC was launched by the honorable minister of health with the purpose of helping the country interrupt transmission of *O. volvulus* and stop MDA by 2025.^20^ As part of its initial work, the NOEC recommended that based on the aforementioned data and subsequent MDA since the 2009 and 2012 surveys, new “stop-MDA” surveys based on the WHO guidelines be conducted in Plateau and Nasarawa states. To facilitate the assessment process, the NOEC recommended the villages where the required entomological and serological assessments should take place. These were to be “first-line villages” based on a desk review of maps that indicated communities close to rapidly flowing rivers, making them most likely to have high vector densities and accordingly at high risk of onchocerciasis transmission. Baseline data on onchocerciasis prevalence and inclusion in the onchocerciasis MDA program were also important considerations for selecting survey sites, but the absence of this information did not exclude sites from being selected. The 33 villages for Plateau and Nasarawa states selected by NOEC are shown in Figure 3. The NOEC further recommended (noting the difficulties in the Evans study with capturing sufficient flies) the use of the Esperanza window trap (EWT) to supplement human landing captures (HLCs) to be able to collect the 6,000 vectors in each state needed to meet the WHO requirements.^21^

## METHODOLOGY

Serological and entomological surveys were conducted from May through October 2017. In addition to the 33 NOEC-selected villages, we also surveyed six SVs, three in each state (Table 2). The mean 1991 baseline skin snip mf prevalence in adult males in these SVs was 64% (range 51–93%). In 2009, the prevalence was 0.1% (range 0–0.27%) among 2,012 adults of both genders.

**Table 2:** Baseline (1991/1992), 2009, and 2017 results in sentinel villages assessed in the Plateau and Nasarawa states stop-mass drug administration surveys in 2017

Sentinel village (local government areas)	Baseline* adult males mf % (1991–1992)	Mf at midpoint, adults examined (2009)	Midpoint mf positive (2009)	Midpoint mf % (2009)	Number of children examined by OV16 ELISA (2017)	Number of OV16 positive (%) (2017)	Number of flies examined by O150 PCR (2017)	Number of annual IV treatment rounds
Nyanji (Toto)	88% (1991)	252	0	0%	165	0	938	25
Bayan Dutse (Akwanga)	93% (1991)	271	1	0.37%	103	0	3,540	26
Angwan Habu (Akwanga)	75% (1992)	337	0	0%	33	0	4,765	25
Bakin Kogi Lemoro (Bassa)	23.3% (1992)	390	0	0%	182	1 (0.55%)	3,956	25
Mafara (Bassa)	51% (1991)	396	0	0%	162	0	84	26
Godong (Jos East)	56% (1991)	366	1	0.27%	157	0	230	26
Total	64%†	2,012	2	0.10%	802	1 (0.12%)	13,513‡	25–26

mf = microfilariae.

* Based on examination of 30–50 male residents/village, the exact number unavailable.

† Mean of percentages.

‡ All head pools were PCR negative for *Onchocerca volvulus* DNA.

### OV16 surveys in children.

Schools in the selected communities were the basis for sampling of children. Participation in the surveys was voluntary. Individual oral assent was obtained from selected students, and written informed consent obtained from each school’s headmaster or his/her representative. Primary school children aged between 5 and 9.5 years were randomly selected from each school. Finger-prick blood samples were obtained by certified laboratory scientists from all verbally assenting children. After cleansing the finger and pricking it with a sterile lancet, the resulting blood that filled the 100-uL capillary tube was immediately placed on Whatman number 2 filter papers (Sigma-Aldrich, St. Louis, MO). The filter papers were air dried, then separated by sheets of papers, and stored in plastic bags in a cooler until they were returned to the Carter Center (TCC) laboratory in Jos, the capital of Plateau state, where they were stored at −20°C. Blood samples were processed for IgG4 antibody against the OV16 recombinant antigen using the standard Onchocerciasis Elimination Program for the Americas (“OEPA”) ELISA methodology as described.^22^

### Entomological survey.

Two HLC sites were established in known or potential local *Simulium* vector–breeding points within 3 km of each selected village. Villagers were asked to identify three to four persons who would serve as attractants and flycatchers. The village teams were supervised by the LGA ministry of health personnel and TCC staff. Fly catches took place during the peak blackfly breeding season, mid-June through August 2017. Each site had four capture days per month, each consisting of hourly collections (50 minutes of catches and 10 minutes of rest) between 7:00 am and 5:50 pm Fly “attractants” exposed their lower limbs and sat quietly with bijou bottles (“tubes”) waiting the vector flies. Flies were collected just after they landed and before they could take a blood meal by placing the tubes over the fly, and then, the tube was gently agitated to make the fly stop its attempt to suck blood. Flies were pooled by transfering to another bottle containing isopropanol. These preservative tubes were labeled with the date, method of capture (HLC), number of flies contained, and village site.

Esperanza window traps were placed within 3 km of known or potential local breeding sites. They were made of 1-m *×* 33 cm blue-colored vinyl tarpaulin stripes flanking a 1-m *×* 33cm black tarpaulin stripe attached to a 1-m^2^ wooden frame.^23^ The surfaces were coated with Tangle-Trap^®^ insect trap coating paste (Contech, Victoria, Canada). The trap was mounted perpendicular to the ground on a stand elevated a few centimeters off the ground. Carbon dioxide was generated from a gallon plastic bottle containing 2 L of water, bakers’ yeast, and sugar.^23^ This solution was replaced every 48 hours. Rubber tubing from the plastic bottle conducted CO_2_ to the top of the trap. Dirty clothes from HLC attractants were hung at the top of the trap to serve as human scent lure. Vector flies stuck to the surface were identified and washed in kerosene to dislodge them from the Tangle-Trap^®^ adhesive. These were collected in a separate tube and preserved in isopropanol. Each collection tube was labeled with the date range of the catch, method of capture (EWT), number of flies contained, and village site.

The tubes were sent to the Jos TCC laboratory where the preserved flies were examined under a dissecting microscope. *Simulium damnosum* s.l. flies so identified were pooled in groups of up to 100 according to the village site. The heads were separated from the bodies by freezing the flies in liquid nitrogen vapor, agitation to shear off the heads, and then sieving them out in a 25 mm mesh. The DNA was extracted from head pools, and an O-150 PCR analysis was conducted as previously described.^24^ The PCR products were detected by the OVS2fl probe detected with an alkaline phosphatase–labeled anti-fluorescein antibody and read using ELISA, also as previously described.^25^

## Statistical Analysis

The surveys were primarily powered to make a WHO stop-MDA decision for each state. We also analyzed the data for the two-state region overall, by endemicity of the survey sites (meso-/hyperendemic and non-/hypo-endemic), and in the SV grouping (Table 2). Criteria for success at interrupting onchocerciasis transmission were both an OV16 antibody prevalence in children of < 0.1% (UCL) and a rate of infective blackflies of < 1/2,000 (UCL). When positive samples were found, the 95% CIs for OV16 antibody prevalence were determined by conventional means using the formula:

$$95\%\;\text{confidence interval}=\text{proportion positive}\left(p\right)\pm 1.96\sqrt{\frac{p\left(1-p\right)}{n}}$$

In cases where no positive children were identified, the 95% UCL was calculated using the Bayesian algorithm in the PoolScreen 2.1 program package as previously described.^26^ The UCL shown in the results is the upper value generated by these methods. The UCL in the O150 blackfly PCR analysis was calculated using PoolScreen 2.1 (available via https://www.soph.uab.edu/faculty/bst/charles_katholi).

## Ethics Statement

The survey protocol was approved by the state ministries of health of Plateau and Nasarawa and the Emory Institutional Review Board, which considered it non-research, as standard monitoring and evaluation for a public health program (Federal Regulations 45 CFR Section 46.102(d)). Children provided oral assent before finger-stick blood was obtained, and their assent was documented on survey forms. Persons acting as human attractants for blackfly catches were told about the personal risks and community benefits of participation and given the option to opt out of participation at any time without repercussions. All attractants were offered a 150 µg/kg dose of ivermectin at the end of the catching period.

## RESULTS

The 39 sites were located in 19 LGAs: in Plateau state, there were 21 survey sites in 11 LGAs and in Nasarawa, state there were 18 sites in eight LGAs. Surveys were conducted in 11 (92%) of 12 meso-/hyperendemic LGAs and eight (44%) of 18 non-/hypo-endemic LGAs. Twenty-six (67%) of the survey sites were classified as meso-/hyperendemic, and 13 sites as non-/hypo-endemic.

### OV16 surveys in children.

A total of 6,262 school children (3,182 in Nasarawa state and 3,080 in Plateau) were tested for OV16 antibody in the 39 sites. A listing of village sample sizes in Nasarawa and Plateau states is given in Tables 3 and 4. Only two children were antibody positive in the two-state area (OV16 prevalence = 0.032%, UCL 0.076%): one in Nasarawa (0.031%, UCL 0.093%) and one in Plateau (0.032%, UCL 0.096%). The positive child in Nasarawa state was an 8-year-old male from a village in hyperendemic Akwanga LGA. The positive child in Plateau was a 9-year-old male resident of the SV Bakin Kogi Lemoro in mesoendemic Bassa LGA. This child was the only positive child found among 802 tested in the six SVs (Table 2). Both positive children are being provided with ivermectin treatment and follow-up. When analyzed by endemicity across the two-state area, 4,199 children were from meso-/hyperendemic LGAs where two children were positive (OV16 prevalence = 0.048%, UCL 0.114%). In non-/hypo-endemic LGAs, there were no OV16 positives among 2,063 children tested, but this result could not statistically exclude 0.1% (OV16 prevalence = 0%, UCL 0.145%).

**Table 3:** Plateau state: 2017 ELISA test results for IgG4 antibody to Ov16 in children (*n* = 3,080), from 21 sites

local government area (baseline endemicity)	Village	Samples received	Samples analyzed	No. of positive results	% positive
Bassa (Meso)	Amokatako	183	183	0	0.00
	Bakin Kogi Lemoro*	182	182	1	0.50
	Mafara*	162	162	0	0.00
	Majaja	91	91	0	0.00
Bokkos (Meso)	Daffo	184	184	0	0.00
Jos East (Meso)	Godong*	157	157	0	0.00
Kanke (Meso)	Jinglai	88	88	0	0.00
Langtang South (Non-Hypo)	Sabon Gida	212	212	0	0.00
Mangu (Non-Hypo)	Fwanko	79	79	0	0.00
Mikang (Non-Hypo)	Lifidi	109	109	0	0.00
	Piapung	200	200	0	0.00
Pankshin (Meso)	Gung	122	122	0	0.00
	Jing	150	150	0	0.00
	Jivir	168	168	0	0.00
Qua’an Pan (Non-Hypo)	Bong	100	100	0	0.00
	Kwalla	205	205	0	0.00
Riyom (Non-Hypo)	Bum	184	184	0	0.00
Shendam (Non-Hypo)	Shimankar	206	206	0	0.00
Wase (Non-Hypo)	Gumshar	194	194	0	0.00
	Lamba	72	72	0	0.00
	Sabongida Mavo	32	32	0	0.00
TOTAL		3,080	3,080	1	0.03

* Sentinel village.

**Table 4:** Nasarawa state: 2017 ELISA test results for IgG4 antibody to Ov16 in children (*n* = 3,182), from 18 sites

local government area (baseline endemicity)	Village	No. of samples received	No. of samples analyzed	No. of positive results	% positive
Akwanga (Hyper)	Akewa	178	178	0	0.00
	Alushi	156	156	1	0.60
	Anguwan Habu*	33	33	0	0.00
	Anguwan Zaria	374	374	0	0.00
	Bayan Dutse*	103	103	0	0.00
	Gbuja	37	37	0	0.00
Awe (Non-Hypo)	Wuse	250	250	0	0.00
Karu (Meso)	Jankanwa	221	221	0	0.00
Kokona (Meso)	Guruku	270	270	0	0.00
	Nindama	86	86	0	0.00
Lafia (Meso)	Arikiya	176	176	0	0.00
	Ugah	229	229	0	0.00
Nasarawa Egon (Meso)	Ezzen Sarki	263	263	0	0.00
Obi (Non-Hypo)	Akuku	220	220	0	0.00
Toto (Hyper)	Kuru	179	179	0	0.00
	Manya	52	52	0	0.00
	Nyanji*	165	165	0	0.00
	Umaisha	190	190	0	0.00
TOTAL		3,182	3,182	1	0.03

* Sentinel village.

### Entomological survey.

A total of 19,056 *S. damnosum* s.l. flies were collected in the 39 surveyed sites. Surveyed sites’ collection results by state are given in Tables 5 and 6. Eight sites (20%) did not produce any vectors, and an additional 10 (26%) produced fewer than 20 vectors. Sentinel villages (Table 2) were the most productive in the survey (13,513 flies, 71%). More flies were captured by HLC (10,452 flies, or 55.3%) than by EWT (8,526, 44.7%). A higher percentage of flies were captured by EWT in Plateau state (85.8%) than in Nasarawa (25.4%, Table 7). The 25 (64%) capture sites in meso-/hyperendemic LGAs contributed 90% (17,071) of the *S. damnosum* captures; only 1,985 were captured from the 13 sites in eight non-/hypo-endemic LGAs (most from Wase LGA, Plateau state).

**Table 5:** Plateau state: 2017 results from O150 PCR analysis of *Simulium damnosum* s.l. heads for *Onchocerca volvulus* DNA, from 21 sites

Local government area (baseline endemicity)	Village	No. of blackflies received	No. of blackflies analyzed	No. of pools of 100	No. of pools < 100*	No. of positives	% positive
Bassa (Meso)	Amokatako	71	71	0	1(71)	0	0
	Bakin Kogi Lemoro†	3,956	3,956	39	1(56)	0	0
	Mafara†	84	84	0	1(84)	0	0
	Majaja	5	5	0	1(5)	0	0
Bokkos (Meso)	Daffo	2	2	0	1(2)	0	0
Jos East (Meso)	Godong†	230	230	2	1(30)	0	0
Kanke (Meso)	Jinglai	53	53	0	1(53)	0	0
Langtang South (non/hypo)	Sabon Gida	31	31	0	1(31)	0	0
Mangu (non/hypo)	Fwanko	9	9	0	1(9)	0	0
Mikang (non/hypo)	Lifidi	83	83	0	1(83)	0	0
	Piapung	98	98	0	1(98)	0	0
Pankshin (Meso)	Gung	11	11	0	1(11)	0	0
	Jing	44	44	0	1(44)	0	0
	Jivir	2	2	0	1(2)	0	0
Qua’an Pan (non/hypo)	Bong	0	0	0	0	0	0
	Kwalla	6	6	0	1(6)	0	0
Riyom (non/hypo)	Bum	12	12	0	1(12)	0	0
Shendam (non/hypo)	Shimankar	0	0	0	0	0	0
Wase (non/hypo)	Gumshar	95	95	0	1(95)	0	0
	Lamba	1,020	1,020	10	1(20)	0	0
	Sabongida Mavo	300	300	3	0	0	0
TOTAL		6,112	6,112	54	18	0	0

* Parentheses show the number of flies in the pool tested.

† Sentinel village.

**Table 6:** Nasarawa state: 2017 results from O150 PCR analysis of *Simulium damnosum* s.l. heads for *Onchocerca volvulus* DNA, from 18 sites

Local government area (baseline endemicity)	Village	No. of blackflies received	No. of blackflies analyzed	No. of pools of 100	No. of pools< 100*	No. of pools positive	% positive
Akwanga (Hyper)	Akewa	6	6	0	1(6)	0	0
	Alushi	1,247	1,247	12	1(47)	0	0
	Anguwan Habu†	4,765	4,765	47	1(65)	0	0
	Anguwan Zaria	1,449	1,449	14	1(49)	0	0
	Bayan Dutse†	3,540	3,540	35	1(40)	0	0
	Gbuja	0	0	0	0	0	0
Awe (non/hypo)	Wuse	0	0	0	0	0	0
Karu (Meso)	Jankanwa	33	33	0	1(33)	0	0
Kokona (Meso)	Guruku	247	247	2	1(47)	0	0
	Nindama	3	3	0	1(3)	0	0
Lafia (Meso)	Arikiya	0	0	0	0	0	0
	Ugah	1	1	0	1	0	0
Nasarawa Egon (Meso)	Ezzen Sarki	384	384	3	1(84)	0	0
Obi (non/hypo)	Adudu	331	331	3	1(31)	0	0
Toto (Hyper)	Kuru	0	0	0	0	0	0
	Manya	0	0	0	0	0	0
	Nyanji†	938	938	9	1(38)	0	0
	Umaisha	0	0	0	0	0	0
TOTAL		12,944	12,944	125	12	0	0

* Parentheses show numbers of flies in the pool tested.

† Sentinel village.

**Table 7:** Method of capture of *Simulium damnosum* sl in Plateau and Nasarawa states in 2017 stop-mass drug administration studies

State	Total flies	EWT		HLC	
	EWT/HLC	Flies	%	Flies	%
Plateau	6,112	5,243	85.8	869	14.2
Nasarawa	12,944	3,283	25.4	9,661	74.6
Total	19,056	8,526	44.7	10,452	55.3

EWT = Esperanza window trap; HLC = human landing capture.

The *S. damnosum* heads were tested by village in 179 pools of 100 and 30 pools of under 100 (Tables 5 and 6). All pools were negative for *O. volvulus* DNA in O-150 PCR, resulting in fulfillment of the WHO stop-MDA criterion of < 1/2,000 infective flies in a combined analysis (UCL 0.20 infective flies/2,000), and individual analyses for Plateau state (UCL 0.63/2,000) and Nasarawa state (UCL 0.30/2,000). Similarly, negative PCR results from the 17,071 flies from meso-/hyperendemic LGAs in the two-state area resulted in < 1/2,000 infective flies (UCL 0.22/2,000). However, there were insufficient flies (1,985) tested from the non-/hypo-endemic areas to meet the WHO entomology criterion (UCL 1.93/2,000).

## DISCUSSION

Plateau and Nasarawa states have individually met the 2016 WHO criteria for interruption of transmission of onchocerciasis and for stopping ivermectin MDA.^17^ Two surveys were conducted, each designed and powered to collect a sample sufficient to allow a stop-MDA decision for each state. Each state demonstrated a 95% UCL of OV16 positivity in children of < 0.1% and a 95% UCL of vector infectivity of < 1/2,000. The NOEC conducted a detailed review of these data at its meeting in December 2017 and recommended to the FMOH that MDA be stopped in the two states and that posttreatment surveillance (PTS) be launched in 2018. The NOEC also recommended that a health information campaign be conducted throughout 2018 to inform the communities in the 12 meso-hyperendemic LGAs of the reason for stopping treatment, noting that MDA in the other 18 non-/hypo-endemic LGAs had been stopped in 2012, with the successful conclusion of the LF treatment program. The FMOH accepted the NOEC recommendation, which resulted in 2.2 million treatments being halted as of January 2018. This was the first “stop-MDA” event for onchocerciasis in Nigeria and to our knowledge is the largest single stop-MDA decision ever for onchocerciasis.

The NOEC sampling sites were selected without a requirement that there be baseline endemicity available for those sites or that they be under the onchocerciasis MDA program. Thus, the MDA programs assessed in the survey included both onchocerciasis meso-/hyperendemic LGAs with long duration of MDA for onchocerciasis (25–26 years, 1992–2017) and onchocerciasis non-/hypo-endemic LGAs with shorter MDA periods administered only during the shorter LF treatment period (8–11 years, 2002–2012). One could debate this approach and argue that the sampling should have been drawn only from the meso-/hyperendemic LGAs. In fact, it was because of this concern that we included six SVs (three in each state) that were among some of the most highly endemic communities identified at the start of the campaign. Given the stringent WHO “stop-MDA” criteria, we believed that elevated positivity in either of the indices in one of those villages would have resulted in the entire state failing the exercise. Further evidence that this study was weighted toward the meso-/hyperendemic areas includes the fact that 92% of meso-/hyperendemic LGAs were sampled (compared with 44% of non-/hypo-endemic LGAs) and two-thirds of the survey sites were in meso-/hyperendemic LGAs. When meso-/hyperendemic numbers were combined for the two states, the entomology criterion was met (UCL 0.22/2,000), and the serology criterion was almost met (two OV16 positive children among 4,199 tested, OV16 prevalence = 0.048%, UCL 0.114%).

Serological data collected from the 13 non-/hypo-endemic LGAs provide suggestive evidence that the relatively short duration of LF MDA was sufficient to eliminate “hypo-endemic” onchocerciasis (defined in this case as a baseline mf prevalence of 1% to < 5%). None of 2,063 children tested had OV16 antibody, but the sample was insufficient to exclude a seroprevalence of 0.1% needed to meet the WHO stop-MDA serological guidelines. However, in an unpublished 2018 study (G. Noland, personal communication, 2019) in the Plateau state LGAs of Mikang and Kanam (the latter LGA was not included in this study), none of 1,561 (6- to 7-year-old) children were OV16 positive on the OV16-Wb123 Biplex rapid diagnostic test (Standard Diagnostics, Suwon, Republic of Korea).^27^ If we were to combine these data with our own from non-/hypo-endemic villages, zero positives among 3,624 children would now be a sufficient sample to exclude 0.1% (0% OV16 prevalence, UCL 0.053%). In our hands, the OEPA Ov16 ELISA compared favorably with a monoplex version of the same RDT (Bioline Ov16 rapid test card, Standard Diagnostics) in another unpublished study of just more than 1,000 DBS samples taken from residents of hypo-endemic onchocerciasis LGAs in southeastern Nigeria. In that study, we found a 99.3% agreement between the two tests, 91.9% agreement among positives and 99.7% agreement among negatives (L. Rakers, personal communication, 2019).

The entomological assessment in these 13 non-/hypo-endemic villages were also negative, but there were insufficient flies tested (1,985) to statistically exclude the < 1/2,000 infective fly threshold.

It is important to note that these findings from the non-/hypo-endemic LGA constituted a PTS survey 5 years after halting (LF) MDA. We might conclude that either onchocerciasis transmission did not originally exist in these areas (because the mf prevalence and vector abundance were very low) or if transmission existed, it was broken by the ivermectin-based MDA provided by the LF program. It is also important to note that the term “hypo-endemicity” used in this report (mf prevalence of 1% to < 5%) considers endemicity levels considerably below the standard APOC “hypo-endemic” definition based on nodule rates. In the APOC/REMO case, hypo-endemicity refers to a nodule prevalence between 5 and < 20%, where the expected corresponding mf prevalence could be as high as 35%.^28,29^ Additional serological studies in children and adults are planned in non-/hypo-endemic onchocerciasis LGAs to better understand these findings.

The 2009 Plateau and Nasarawa onchocerciasis impact survey could not obtain sufficient numbers of vectors by HLC to satisfy the WHO entomological requirements.^11^ An important recommendation from that work was to find new ways to improve *S. damnosum* captures without increasing the time required by human attractants and field personnel. The challenge of collecting sufficient flies was also noted in impact studies in Nigeria conducted by APOC in neighboring Kaduna state and has been an issue for other countries as well.^30,31^ The EWTs variably increased captures by up to 50% over HLC; without the traps, we might have repeated our 2009 experience and failed to meet this key criterion of the WHO stop-MDA guidelines. A major advance for Nigeria was the NOEC decision to accept results from PCR testing of trapped vectors as acceptable entomological evidence in stop-MDA surveys in Nigeria.

The next challenge for Plateau and Nasarawa states will be to conduct a thorough PTS to monitor for reintroduction of *O. volvulus* transmission. Given the slow evolution of mf in skin and the OV16 response in incident infections, the earliest signal for reintroduction of onchocerciasis transmission would be positive PCR pools from vector blackflies.^17,32^ The NOEC expressed greatest concern about reintroduction of onchocerciasis from neighboring states through movement of infected humans or vectors. The plan is for PCR monitoring of pools of *S. damnosum* s.l. captured from new HLC and EWT sites on or very close to the borders, especially with states with ongoing transmission; Taraba state (to the east) and Benue state (to the south).

The duration of this annual MDA program was 25–26 years. This is in line with predictions by Winnen et al.,^33^ whose models showed that in most areas with meso-/hyperendemic onchocerciasis annual MDA could only eliminate transmission after about 27 years. More recent experience and models suggest that with annual MDA elimination can be achieved in 14–17 years.^34,35^ Considering the extremely low microfilaria prevalence (< 1%) and OV16 rates in children (UCL 0.32%) found in the 2009 Plateau Nasarawa impact survey,^8^ one could imagine that the WHO stop-MDA thresholds could have been successfully met in 2012 or 2013, after an additional 3–4 years of treatment.

It was a major challenge for the Plateau and Nasarawa programs to meet the WHO serological threshold of 0.1% with 95% confidence. In each state, a single positive child of the recommended 3,000 threatened to fail the serological break point OV16 threshold.^36^ This study points to the need for WHO to reexamine its stop-MDA serological thresholds that are difficult to measure and likely to be overly conservative. The WHO established the Onchocerciasis Technical Advisory Subgroup (OTS) in 2017 to help develop an operational research strategy to develop new, evidence-based guidelines, including raising the serological threshold for stop MDA decisions. Models, test parameters, sampling strategies, and statistics are being included in OTS considerations,^37,38^ and the general consensus is that the serological threshold should be considerably increased by at least an order of magnitude. Until the OV16 thresholds are adjusted, however, one important interim recommendation from this study is to assure that in all future OV16 stop-MDA studies, laboratories are trained and able to test OV16-positive children by skin snip PCR. The 2016 WHO guidelines permit up to nine OV16-positive/PCR skin snip–negative children before including OV16-positive children in the UCL tallies.^17^ If the two OV16-positive children in this study had been tested and determined to be PCR negative, the state UCL calculations would not have so closely approached 0.1%, and the decisions for stopping MDA in Plateau and Nasarawa states would have been much more comfortable to make.
